# Central obesity and radiographic severity are associated with symptomatic hand osteoarthritis: a population-based cross-sectional study

**DOI:** 10.1007/s00296-025-05891-7

**Published:** 2025-05-17

**Authors:** Sofia Pimenta, Hernâni Gonçalves, Madalena Pimenta, Ana Martins, Lúcia Costa, Tiago Guimarães, Ana Rodrigues, Raquel Lucas

**Affiliations:** 1 Department of Rheumatology, Centro Hospitalar Universitário São João, Alameda Professor Hernâni Monteiro, 4200-319 Porto, Portugal; 2 Faculty of Medicine, University of Porto, Porto, Portugal; 3 CINTESIS – Center for Health Technology and Services Research, Faculty of Medicine, University of Porto, Porto, Portugal; 4 Department of Community Medicine, Information and Health Decision Sciences, Faculty of Medicine, University of Porto, Porto, Portugal; 5 Department of Radiology, Centro Clínico Universitário D. Pedro V, Armed Forces Hospital, Porto, Portugal; 6 Department of Clinical Pathology, Centro Hospitalar Universitário São João, Porto, Portugal; 7 Comprehensive Health Research Centre (CHRC), Faculty of Medical Sciences, NOVA Medical School, NOVA University of Lisbon, Lisbon, Portugal; 8 Epidemiology Research Unit, Institute of Public Health, University of Porto, Porto, Portugal

**Keywords:** Hand joints, Osteoarthritis, Pain, Central obesity, Radiography, Aging

## Abstract

To investigate the association between cardiometabolic factors, obesity, radiographic severity, and symptomatic hand osteoarthritis (HOA), as the role of these factors in HOA remains unclear. A cross-sectional analysis in the EPIPorto cohort included participants with HOA (≥ 1 joint with Kellgren-Lawrence (KL) grade ≥ 2 and/or American College of Rheumatology criteria). Cardiovascular risk factors, anthropometric measures, and radiographic severity (sum of KL hand score [0–128]) and number of affected joints [0–32]) were analysed. We tested the association between these factors and symptomatic HOA (≥ 1 joint with KL ≥ 2 and hand pain in the last month) by multivariable logistic regression. Of the 858 participants with HOA (61% women, mean age 59.6 years), 807 met radiographic criteria, and 160 presented symptomatic HOA. Among these, 77% were overweight or obese, 81% hypertensive, 95% had dyslipidaemia, and 20% were diabetic. Body mass index, waist circumference, and waist-to-height ratio, were associated with symptomatic HOA (OR 1.04, 95% CI 1.00; 1.09), (OR 1.02, 95% CI 1.00; 1.04), (OR 1.03, 95% CI 1.01; 1.06). Diabetes, hypertension, and dyslipidaemia showed no association. We observed an association between the KL score, the number of affected joints, and symptomatic HOA (OR 1.09, 95% CI 1.07; 1.12), (OR 1.09, 95% CI 1.06; 1.12). Increased central obesity and radiographic severity are associated with symptomatic HOA, highlighting the potential role of adiposity in HOA pain. These findings underscore the importance of weight management to improve pain outcomes in HOA. Furthermore, assessing radiographic changes may aid monitoring of disease symptoms. Further studies are needed to validate these associations and inform evidence-based clinical practice.

## Introduction

Osteoarthritis (OA) is a highly prevalent osteoarticular disease that affects more than 500 million people worldwide [1] and is one of the main causes of functional disability in middle-aged adults and the elderly. As life expectancy increases, it is estimated that the growing incidence of OA will place a major burden on society and healthcare systems [2–4]. OA can involve any joint and is known to affect all joint structures, where cartilage degradation, subchondral bone remodelling and synovial inflammation are observed [5, 6]. The knee is the most affected joint, followed by the hands and hips [1, 7].

Estimates of the prevalence of hand OA (HOA) can vary according to the definition used, as well as sex, age and geographical location of the population studied. It is known that the worldwide prevalence of symptomatic HOA is lower (3–16%) than radiographic HOA (21–92%) [8, 9]. HOA is associated with pain, stiffness, functional limitations, loss of grip strength, aesthetic discomfort and, above all, a reduced quality of life [10]. Its definition is a challenge, as it can be classified in various ways: according to the ACR 1990 clinical criteria [11], by radiographic structural changes (radiographic HOA) or by radiographic changes associated with the presence of typical symptoms (pain or stiffness) referred to as symptomatic HOA [10] and also by the recommendations of the European League Against Rheumatism (EULAR) [12, 13]. Despite this, the importance of interpreting radiographs in the assessment of hand OA remains unclear. There are limited studies exploring the association between radiographic scores, the number of affected joints and pain in hand OA [14]. This raises the question of whether there is any relationship between the extent of radiographic findings and pain in hand OA.

Although the aetiology of HOA is still not well understood, it is known to have a heterogeneous clinical presentation. Symptomatic HOA is particularly noteworthy, as it is one of the subgroups that frequently prompts medical consultation. Given that HOA lacks disease-modifying treatments, underscoring the urgent need for therapeutic advancements and preventive strategies to improve patient outcomes and disease management.

Some risk factors for hand OA have been pointed out, namely being female, over 40 years of age, menopause, genetic factors, ethnicity, obesity, occupation-related use, joint hypermobility, trauma, diet, smoking and alcohol consumption [8, 12, 15–17]. Several researchers have tested to find associations between HOA and certain comorbidities and evidence has been shown, namely the association between cardiovascular disease (CVD) and symptomatic HOA, diabetes mellitus (DM) and pain in HOA and metabolic syndrome and HOA [18–24]. These associations suggest the presence of common underlying systemic mechanisms, particularly in symptomatic HOA. However, conflicting findings also exist, highlighting the complexity of these relationships and the need for further research. Identifying potentially modifiable risk factors for symptomatic HOA may improve disease management and contribute to a more personalized treatment approach.

Our aim was to study the sociodemographic, clinical and radiographic characteristics of HOA in a population-based cohort and to investigate whether cardiometabolic factors, obesity, and radiographic severity are associated with symptomatic HOA.

## Methods

### Study design and population

This is a cross-sectional analysis from a population-based cohort, EPIPorto, and has been reported in accordance with the Strengthening the Reporting of Observational Studies in Epidemiology (STROBE) guidelines for observational studies (https://www.strobe-statement.org). The study started in 1999 when adult community dwellers in the city of Porto, Portugal, were recruited by random digit dialling with households as the sampling frame. Once a household was selected, all residents were identified, and one adult resident was randomly selected as the respondent, without allowing for replacements. The response rate was 70%, resulting in a total of 2485 participants. Participants had regular follow-up assessments with a questionnaire on social, demographic, behavioural and clinical data, as well as a physical examination, including blood sampling. This study is based on a cross-sectional analysis of the 2005–2008 follow-up wave with 1681 participants. Data were extracted on sociodemographic characteristics, anthropometric data, health-related behaviours, hand pain questionnaire, comorbidities and cardiovascular diseases. Data on systemic inflammation and radiographic data were also extracted.

### Participants

This study included EPIPorto participants of the 2005–2008 follow-up wave with ≥ 18 years and with HOA, defined by the ACR clinical criteria [11] and/or radiographic Kellgren-Lawrence (KL) [25] grade ≥ 2 in at least one joint.

Patients with a self-reported or diagnosis by a rheumatologist of rheumatoid arthritis, psoriatic arthritis, spondyloarthropathy, microcrystalline arthropathy, psoriasis, family history of psoriasis, hemochromatosis and trauma to the hand were excluded.

### Data collection

#### Radiographic assessment and definitions

All the bilateral posteroanterior radiographs of the hands in the database were assessed by two expert readers: a radiologist specialised in musculoskeletal diseases and a rheumatologist. The radiographic changes in each joint of both hands were scored using the KL score, which ranged from grade 0 to 4 (grade 0: no OA; grade 1: doubtful OA; grade 2: minimal OA; grade 3: moderate OA; grade 4: severe OA). The following joints were classified: distal interphalangeals (DIPs 2–5), proximal interphalangeals (PIPs 2–5), first interphalangeal (IP-1), metacarpophalangeals (MCPs 1–5), first carpometacarpal and scaphotrapezial. The sum of the KL score ranged from 0 to 128. The inter-observer reliability (intraclass correlation coefficient (ICC), two-way mixed-effects model, absolute agreement) for the sum KL hand score was 0.94. The presence of erosions was determined using phase E (erosive phase) or R (remodelling phase) of the Verbruggen-Veys (VV) anatomical score [26], which was used to assess the same joints.

#### Definition of symptomatic hand osteoarthritis (symptomatic HOA)

Self-reported non-traumatic pain was assessed using a standardised question: "Have you had pain in your hands in the last month?" classified as yes/no.

Participants who had hand pain in the last month and who met the radiographic criteria for HOA (KL grade ≥ 2 in at least one joint) were defined as having symptomatic HOA.

#### Definition of asymptomatic hand osteoarthritis (asymptomatic HOA)

Participants who met the radiographic criteria for HOA (KL grade ≥ 2 in at least one joint), showed no erosions (phase E or R) according to the VV classification, and had not experienced hand pain in the last month were classified as having asymptomatic HOA.

#### Definition of radiographic severity

Radiographic severity was defined by two parameters: the sum of the KL score and the number of joints involved. The sum of the KL score was calculated by adding up the degree of OA in each hand joint, scoring from 0 to 128, with 128 being the worst. The number of hand joints (from 0 to 32) with radiographic OA (KL score ≥ 2) was also calculated.

#### Clinical data

Demographic data such as age, sex, education and occupation were extracted. Education was recorded as completed years of schooling and occupations were classified according to the National Classification of Occupations (Instituto Nacional de Estatística, 2011). The occupations were then categorised into three different classes: low (blue-collar: farmers, skilled and unskilled workers, craftsmen, machine operators and assembly workers), intermediate (lower white-collar: administrative and related workers, service and sales workers), and high (upper white-collar: executive civil servants, industrial directors, scientists, middle management and technicians).

Anthropometric data, such as body mass index (BMI) in continuous and categorical variables (BMI < 25, normal weight; BMI 25–29.9, overweight; BMI ≥ 30.0, obesity) [27], waist circumference (WC), waist-to-hip ratio (WHR), waist-to-height ratio (WHtR) and waist-to-height ratio ≥ 0.5, were also used or computed. Anthropometric measurements were conducted by trained professionals following standard procedures. Weight was assessed with participants barefoot and in light clothing, using a digital scale to the nearest 0.1 kg. Height was measured with individuals standing barefoot, heels together, and back against the wall of the stadiometer, with the head positioned according to the Frankfurt plane. Body mass index was calculated by dividing weight (kg) by the height (m) squared. The WC was determined at approximately halfway between the lowest margin of the last palpable rib and the top of the iliac. The hip circumference was defined as the perimeter surrounding the widest part of the buttocks at the axial plane. The WHR was calculated by dividing the waist circumference (cm) by the hip circumference (cm), and the WHtR was calculated by dividing the waist circumference (cm) by the height (cm).

We also collected data on behavioural variables such as the practice of exercise (current regular practice of any sport or exercise, yes/no), alcohol consumption (ever vs never) tobacco consumption (current smoker, former smoker over 6 months or never smoked), data on non-traumatic hand pain through a standardized question (“Have you had hand pain in the last month?” yes/no) and also, among women, menopausal status.

Diabetes was defined as self-reported history of a diabetes diagnosis or measured fasting blood glucose ≥ 126 mg/dl [28] or taking oral antidiabetics/insulin. Hypertension was defined as self-reported or assessed systolic blood pressure (BP) ≥ 140 mmHg and/or diastolic BP ≥ 90 mmHg [29] or by use of antihypertensive medication. Dyslipidaemia was defined as a self-reported previous diagnosis or by total cholesterol ≥ 200 mg/dl or LDL ≥ 116 mg/dl or triglycerides ≥ 150 mg/dl [30–32] or taking medication for dyslipidaemia. Data on cardiovascular diseases were self-reported as ever been diagnosed by a doctor with one of the following: myocardial infarction, angina, heart failure, arrhythmia, or stroke.

Data on high-sensitivity C-reactive protein (hsCRP) was obtained. Blood was sampled after a 12 h overnight fast. Serum samples were stored at − 80 °C before analysis. High-sensitivity CRP concentrations were determined through particle-enhanced immunonephelometry hsCRP Flex® reagent cartridge (Siemens, Lisbon, Portugal), using a Dimension Vista® 500 automated analyser (Siemens, Lisbon, Portugal). The assay is sufficiently sensitive to detect 0.2 mg/l.

### Statistical analysis

Descriptive statistics for continuous variables were presented as mean and standard deviation. Categorical variables were summarized using absolute and relative frequencies. The t-test was used to compare continuous variables with a normal distribution, while the Pearson's Chi-Square Test for Independence or Fisher's exact test was employed for categorical variables. The Mann–Whitney U test was utilized to analyse BMI categories. Logistic regression analysis was conducted to quantify the association between the studied independent variables (sociodemographic and anthropometric data, behavioural variables, menopause, comorbidities and cardiovascular disease, systemic inflammation and radiographic data) and symptomatic HOA (dependent variable). All odds ratios (OR) in the multivariable model were adjusted for age, sex and education. Data analysis was performed using R version 4.3.2 and RStudio.

## Results

**Fig. 1:**
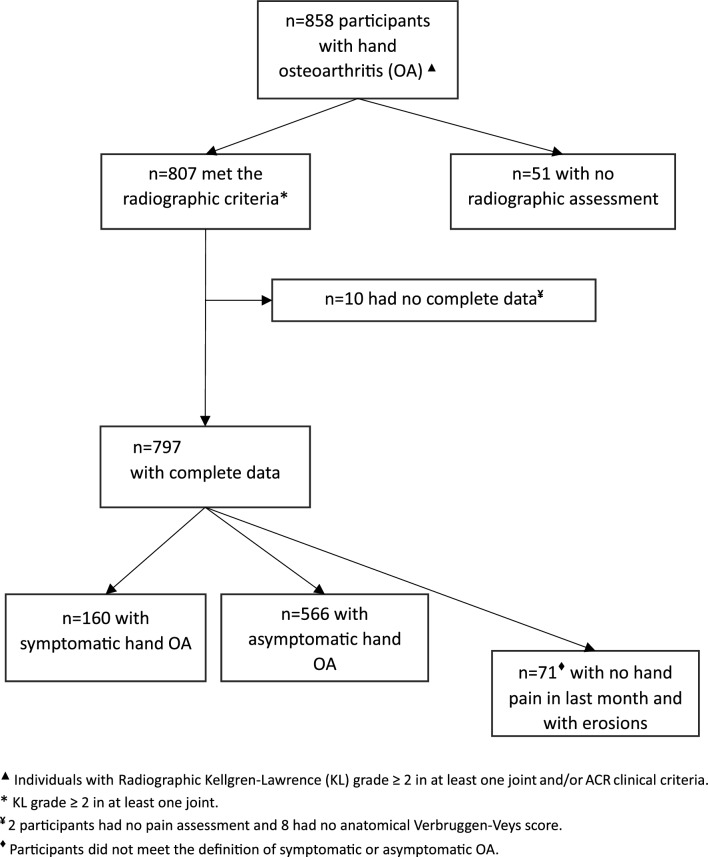
Flowchart of the Individuals with hand osteoarthritis included in cross-sectional analysis

Of the 1681 EPIPorto participants of the wave 2005–2008, 887 had a radiographic assessment of both hands. Of these, 35 did not have radiographic criteria for HOA and 45 were excluded (10 with psoriasis and 35 with chronic inflammatory joint diseases). Of all the participants, 264 had a clinical assessment of the hand, of which 150 met the ACR clinical criteria (51 had no radiographic assessment due to equipment unavailability). Therefore, 858 individuals with hand osteoarthritis were included in our cross-sectional analysis: 807 had radiographic criteria (KL grade ≥ 2 in at least one joint) of which 2 had no pain assessment and 8 had no anatomical VV score. Of these, 160 (20%) had symptomatic HOA and 566 people had asymptomatic HOA (Fig. 1).

### Sociodemographic, anthropometric, behavioural characteristics, comorbidities, cardiovascular diseases and radiographic severity of patients with HOA

Table 1 shows the descriptive analysis of the participants with HOA. The mean age was 59.6 (13.5) years, 61.3% were women.

**Table 1:** Clinical and radiographic characteristics of patients with hand osteoarthritis (OA)

	Hand osteoarthritis n = 858
**Sociodemographic data**	
Age (years), mean (SD)	59.6 (13.5)
Sex	
Women, n (%)	526 (61.3)
Men, n (%)	332 (38.7)
Education (years) mean (SD)	8.6 (4.9)
Blue-collar occupation, n (%)	17 (1.9)
**Anthropometric data**	
BMI (kg/m^2^), mean (SD)	27.5 (4.8)
< 25, n (%)	258 (30.1)
25–29.9, n (%)	381 (44.4)
≥ 30.0, n (%)	219 (25.5)
Waist circumference (cm), mean (SD)	92.9 (11.8)
Waist-to-Hip Ratio, mean (SD)	0.92 (0.08)
Waist-to-Height Ratio, mean (SD)	0.58 (0.08)
Waist-to-Height Ratio ≥ 0.5, n (%)	745 (86.9)
**Behavioural variables**	
Ever smoker (yes/no) (n = 857), n (%)	366 (42.7)
Alcohol (ever/never) (n = 852), n (%)	717 (84.2)
Physical exercise (yes/no) (n = 857), n (%)	406 (47.4)
**Menopause** (12 months after the final menstrual period) (n = 525), n (%)	402 (76.6)
**Comorbidities and cardiovascular diseases**	
Diabetes (n = 538), n (%)	113 (21)
Hypertension (n = 608), n (%)	490 (80.6)
Dyslipidaemia (n = 784), n (%)	740 (94.4)
Myocardial infarction (n = 857), n (%)	23 (2.7)
Angina pectoris (n = 857), n (%)	46 (5.4)
Congestive heart failure (n = 855), n (%)	28 (3.3)
Cardiac arrhythmia (n = 858), n (%)	115 (13.4)
Stroke (n = 856), n (%)	23 (2.7)
**Systemic inflammation**	
hsCRP, (mg/dl), mean (SD)	0.4 (0.9)
**Radiographic data**	
Sum of hand KL score (0–128), mean (SD)	45.5 (11.2)
Number of joints with KL ≥ 2, (0–32) mean (SD)	11.8 (8.3)

*SD* standard deviation, *BMI* body mass index, *hsCRP* high sensitivity C-Reactive Protein, *KL* Kellgren-Lawrence

The mean BMI was 27.5 (4.8) kg/m^2^ and 70% of participants (n = 600) were overweight or obese. The mean waist circumference was 92.9 (11.8) cm, the mean waist-to-hip ratio was 0.92 (0.08), the waist-to-height ratio was 0.58 (0.08) and 745 participants (87%) had a waist-to-height ratio ≥ 0.5.

It was found that 21% were diabetic, 94.4% had dyslipidaemia and 80.6% were hypertensive.

The mean sum of the KL score was 45.5 (11.2) and the mean number of affected joints with a KL score ≥ 2 was 11.8 (8.3).

### Comparison of sociodemographic, anthropometric, behavioural characteristics, comorbidities, cardiovascular disease and radiographic data between patients with symptomatic and asymptomatic HOA

Of the 797 participants with radiographic osteoarthritis assessed for pain, 160 (20%) were symptomatic and 566 (71%) were asymptomatic. Symptomatic participants had a significantly higher mean age (64.6 vs. 55.9, p < 0.001). There were statistically significant differences in the distributions by sex and menopausal status, with women and postmenopausal women being more common among participants with symptomatic HOA (83.1% vs. 53.7%, p < 0.001 and 93.2% vs. 63.2%, p < 0.001, respectively) (Table 2).

**Table 2:** Comparison of clinical and radiographic characteristics between patients with symptomatic and asymptomatic hand osteoarthritis (HOA)

	Symptomatic OA	Asymptomatic OA	p value
	n = 160	n = 566	
**Sociodemographic data**			
Age (years), mean (SD)	64.6 (9.3)	55.9 (13.8)	** < 0.001**
Sex			^a^** < 0.001**
Women, n (%)	133 (83.1)	304 (53.7)	
Men, n (%)	27 (16.9)	262 (46.3)	
Education (years), mean (SD)	6.8 (4.4)	9.6 (4.9)	** < 0.001**
Blue-collar occupation, n (%)	5 (3.1)	8 (1.4)	^b^0.176
**Anthropometric data**			
BMI (kg/m^2^), mean (SD)	28.8 (5.3)	26.8 (4.6)	** < 0.001**
BMI classes			^c^ < **0.001**
< 25, n (%)	37 (23.1)	194 (34.3)	
25–29.9, n (%)	64 (40.0)	256 (45.2)	
≥ 30.0, n (%)	59 (36.9)	116 (20.5)	
Waist circumference (cm), mean (SD)	94.9 (11.9)	91.7 (11.8)	**0.003**
Waist-to-Hip Ratio, mean (SD)	0.91 (0.07)	0.91 (0.08)	0.815
Waist-to-Height Ratio, mean (SD)	0.61 (0.08)	0.57 (0.07)	** < 0.001**
Waist-to-Height Ratio ≥ 0.5, n (%)	149 (93.1)	468 (82.8)	^a^**0.002**
**Behavioural variables**			
Ever smoker (yes/no), n (%)	39 (24.5)	284 (50,2)	^a^**0.001**
Tobacco classes			** < 0.001**
Current smoker, n (%)	16 (10.0)	120 (21.3)	
Former smoker, n (%)	23 (14.5)	164 (28.9)	
Never smoker, n (%)	120 (75.5)	282 (49.8)	
Alcohol (ever/never), n (%)	131 (83.4)	474 (83.9)	^a^0.903
Physical exercise (yes/no), n (%)	61 (38.4)	283 (50.0)	^a^**0.011**
**Menopause**, n (%)	124 (93.2)	192 (63.2)	^a^** < 0.001**
**Comorbidities and cardiovascular diseases**			
Diabetes, n (%)	24 (19.7)	56 (18.3)	^a^0.791
Hypertension, n (%)	103 (80.5)	282 (78.1)	^a^0.629
Dyslipidaemia, n (%)	145 (95.4)	476 (94.4)	^a^0.693
Cardiovascular diseases in total, n (%)	42 (26.3)	96 (16.9)	^a^**0.008**
Myocardial infarction, n (%)	5 (3.1)	15 (2.6)	^b^0.792
Angina pectoris, n (%)	14 (8.8)	18 (3.2)	^b^**0.002**
Congestive heart failure, n (%)	8 (5.0)	15 (2.6)	^b^0.194
Cardiac arrhythmia, n (%)	31 (19.4)	60 (10.6)	^b^ < **0.001**
Stroke, n (%)	7 (4.4)	10 (1.8)	^b^ 0.076
**Systemic inflammation**			
hsCRP, (mg/dl), mean (SD)	0.44 (1.11)	0.40 (0.86)	0.633
**Radiographic data**, mean (SD)			
Sum of hand KL score (0–128)	52.1 (12.6)	41.7 (7.7)	** < 0.001**
Number of joints with KL ≥ 2, (0–32)	16.3 (8.5)	9.3 (6.9)	** < 0.001**

Statistically significant results are in bold

*BMI* body mass index, *hsCRP* high sensitivity C-Reactive Protein, *KL* Kellgren-Lawrence

^a^Chi-squared test

^b^Fisher's test

^c^Mann-Whitney-U test

Mean BMI was significantly higher in symptomatic HOA participants (28.8 vs. 26.8, p < 0.001). Similarly, the mean waist circumference and the mean waist-to-height ratio were significantly higher in symptomatic participants (94.9 vs. 91.7, p = 0.003 and 0.61 vs. 0.57; p < 0.001).

There were no differences between the groups in terms of diabetes, hypertension and dyslipidaemia. However, symptomatic participants had a higher frequency of angina (8.8% vs. 3.2%, p = 0.002) and arrhythmia (19.4% vs. 10.6%, p < 0.001).

Participants with symptomatic HOA had a significantly higher mean sum KL score than the asymptomatic (52.1 vs 41.7, p =  < 0.001), as well as a higher number of joints with KL ≥ 2 (16.3 vs 9.3, p =  < 0.001) (Table 2).

### Factors associated with symptomatic HOA

#### Sociodemographic and anthropometric factors, behavioural characteristics and menopause

In a multivariable analysis adjusted for age, sex and education we found that BMI (as a continuous variable), WC and WHtR remained associated with symptomatic HOA (OR 1.04, 95% CI 1.00; 1.09), (OR 1.02, 95% CI 1.00; 1.04) and (OR 1.03, 95% CI 1.01; 1.06) respectively (Table 3). Menopause was also associated with symptomatic HOA (OR 2.70, 95% CI 1.05; 6.94).

**Table 3:** Logistic regression analysis of factors associated with symptomatic hand osteoarthritis

	Univariable analysis Crude OR (95% CI)	p value	Multivariable analysis Adjusted* OR (95% CI)	p value
**Sociodemographic data**				
Age (per year increase)	1.06 (1.04, 1.07)	** < 0.001**		
Sex (Men vs Women)	0.24 (0.15, 0.37)	** < 0.001**		
Education (per schooling year)	0.88 (0.84, 0.92)	** < 0.001**		
Blue-collar occupation	2.25 (0.73, 6.98)	0.16	2.87 (0.96, 8.57)	0.059
**Anthropometric data**				
BMI (kg/m^2^)	1.08 (1.04, 1.12)	** < 0.001**	1.04 (1.00, 1.09)	**0.039**
BMI < 25	REF	REF	REF	REF
BMI: 25–29.9	1.31 (0.84, 2.05)	0.234	1.15 (0.70, 1.89)	0.576
BMI ≥ 30.0	2.67 (1.67, 4.27)	** < 0.001**	1.55 (0.91, 2.62)	0.103
Waist circumference (per cm)	1.02 (1.01, 1.04)	**0.003**	1.02 (1.00, 1.04)	**0.034**
Waist-to-Hip Ratio^¥^	^**¥**^1.00 (0.98, 1.02)	0.814	^**¥**^1.01 (0.98, 1.05)	0.397
Waist-to-Height Ratio^¥^	^**¥**^1.08 (1.05, 1.10)	** < 0.001**	^**¥**^1.03 (1.01, 1.06)	**0.019**
Waist-to-Height Ratio < 0.5	REF	REF	REF	REF
Waist-to-Height Ratio ≥ 0.5	2.81 (1.47, 5.38)	**0.002**	1.37 (0.67, 2.81)	0.392
**Behavioural variables**				
Ever smoker (yes/no)	0.32 (0.22, 0.48)	** < 0.001**	0.84 (0.53, 1.35)	0.47
Current smoker	0.31 (0.18, 0.55)	** < 0.001**	0.84 (0.53, 1.35)	0.47
Former smoker	0.33 (0.20, 0.54)	** < 0.001**	0.73 (0.41, 1.30)	0.279
Never smoker	REF	REF	REF	REF
Alcohol consumption (ever/never)	0.97 (0.6, 1.56)	0.891	1.36 (0.81, 2.27)	0.241
Physical exercise (yes/no)	0.62 (0.43, 0.89)	**0.01**	0.82 (0.55, 1.23)	0.333
**Menopause**	8.04 (3.93, 16.44)	** < 0.001**	2.70 (1.05, 6.94)	**0.039**
**Comorbidities and cardiovascular diseases** (yes/no)				
Diabetes	1.09 (0.64, 1.86)	0.743	1.19 (0.67, 2.10)	0.559
Hypertension	1.15 (0.7, 1.91)	0.576	1.07 (0.60, 1.89)	0.816
Dyslipidaemia	1.22 (0.52, 2.85)	0.648	1.21 (0.49, 2.98)	0.675
Cardiovascular diseases in total	1.74 (1.15, 2.64)	**0.009**	1.28 (0.8, 2.06)	0.301
Myocardial infarction	1.18 (0.42, 3.31)	0.746	0.93 (0.36, 2.45)	0.892
Angina pectoris	2.94 (1.43, 6.05)	**0.003**	2.10 (0.94, 4.69)	0.069
Congestive heart failure	1.94 (0.81, 4.67)	0.138	0.93 (0.38, 2.273)	0.874
Cardiac arrhythmia	2.03 (1.26, 3.26)	**0.004**	1.43 (0.83, 2.46)	0.201
Stroke	2.54 (0.95, 6.78)	0.063	1.83 (0.66, 5.05)	0.247
**Systemic inflammation**				
hsCRP, (mg/dl)	1.051 (0.879,1.256)	0.587	1.07 (0.82, 1.41)	0.603
**Radiographic data**				
Sum of hand KL score (0–128)	1.11 (1.09, 1.14)	** < 0.001**	1.09 (1.07, 1.12)	** < 0.001**
Number of joints with KL ≥ 2, (0–32)	1.12 (1.09, 1.14)	** < 0.001**	1.09 (1.06, 1.12)	** < 0.001**

Statistically significant results are in bold

*OR* odds ratio, *CI* confidence interval, *BMI* body mass index, *hsCRP* high sensitivity C-Reactive Protein; *KL* Kellgren-Lawrence

^*^OR adjusted for age, sex and education

^**¥**^Per hundredth of variation

#### Comorbidities, cardiovascular diseases and systemic inflammation

In multivariable analysis adjusted for age, sex and education, angina was associated with symptomatic HOA (OR 2.10, 95% CI 0.94, 4.69), but was not significant (p = 0.069) (Table 3).

#### Radiographic severity

Both univariable and multivariable analyses showed that the sum of the KL score and the number of joints with KL ≥ 2 were positively associated with symptomatic HOA (adjusted OR 1.09, 95% CI 1.07, 1.12) and (adjusted OR 1.09, 95% CI 1.06, 1.12), respectively (Table 3).

## Discussion

This study demonstrated an association between BMI, WC, WHtR, and symptomatic HOA. These findings reinforce the link between BMI and hand OA, while highlighting the importance of WC and WHtR as indicators of central adiposity in symptomatic HOA. Furthermore, individuals with hand osteoarthritis in this population showed higher frequencies of cardiovascular risk factors, including dyslipidaemia, hypertension, overweight/obesity, and diabetes, compared to the general Portuguese population [32–34].

It is known that anthropometric measurements, namely WC and WHtR, are used to assess central adiposity [35]. When measuring central obesity, it is important to consider that the BMI sometimes fails to specify whether a high BMI is related to abdominal obesity, as BMI does not differentiate between the sources of weight (fat or muscle) nor distinguish between peripheral and central obesity (fat distribution). A study from the US National Health and Nutrition Examination Survey (NHANES) 1999–2004 found that 33.1% of men and 51.9% of women had abdominal obesity despite having a BMI in the healthy range [36]. Therefore, the authors decided to assess anthropometric measurements other than BMI in this study.

Furthermore, numerous studies have highlighted the significance of WC and WHtR as predictors of cardiovascular (CVD), metabolic and chronic diseases [37–40]. Dezfouli et al. [41] demonstrated that higher WHtR values were associated with an increased risk of CVD and overall mortality. In addition to its continuous measurement, the threshold value of 0.5 is also indicated as the cutoff point beyond which there is an increased health risk. One of the advantages of WHtR over other measurements of central fat is that it is independent of age, sex or ethnicity [40].

The results of this study showed a positive association between numerical WHtR and symptomatic HOA and between WC and symptomatic HOA. However, we found no association with WHtR ≥ 0.5 in multivariable regression analysis, likely because most participants with hand OA in our study already had a high WHtR. These findings highlight the significance of central obesity as a potential contributor to symptomatic HOA, suggesting the importance of metabolic/systemic factors as a potentially modifiable risk factor.

In a multivariable cross-sectional analysis, we also found a significant association between BMI and symptomatic hand OA. This association has been the subject of several publications. Yusuf et al*.* showed a twofold higher risk of hand OA in obese people than in non-obese people [42]. Another study showed that a higher BMI was associated with greater intensity of hand pain and that the systemic effects of obesity could play a greater mediating role in hand pain than in lower extremity pain [43]. The association between obesity and hand OA has been explained by the ability of the adipocyte to produce adipokines and pro-inflammatory cytokines that can contribute to the pathogenesis and pain of OA [44–46]. The secretion of these mediators will depend on the type of adipose tissue, with visceral fat being more actively secretory than subcutaneous fat [46–48]. A meta-analysis also showed that BMI was positively associated with hand OA, but the interpretation of these results was hampered by differences in methodology and definition of OA [49]. Our results confirm an association between BMI and symptomatic HOA, as well as between increased central obesity and symptomatic HOA. These data emphasize the potential role of adiposity in the manifestation of the disease.

This study also found an association between angina and symptomatic HOA, although not significant. The association between osteoarthritis and CVD has been studied by various authors [18–20, 50, 51]. This association has been explained by a number of reasons, including the existence of risk factors common to both OA and CVD, the fact that chronic low-grade inflammation may be one of the main factors linking CVD to OA, and the fact that changes to the extracellular matrix found in OA may increase the risk of CVD even more [19, 52, 53]. Hall et al*.* demonstrated in a meta-analysis that there was a statistically significant increase in heart failure and ischemic heart disease in individuals with OA compared to controls without osteoarthritis [20]. Veronese et al*.* concluded that OA, especially hand OA in women, was statistically associated with a higher risk of developing CVD [19]. In contrast, Hoeven et al*.* found that patients with OA did not present a statistically increased risk of CVD, particularly after adjustment for disability [50].

Haugen et al. observed that symptomatic HOA, but not radiographic hand OA, was associated with an increased risk of coronary heart disease events [51]. Our results are in line with this study. Interestingly, similar to these authors, we did not find an association with heart failure or stroke. We may consider whether there is variability in the contribution of different risk factors to individual CVD outcomes or if there are distinct pathogenic processes [51]. Recently, Courties et al*.* showed that symptomatic HOA and worse HOA clinical course were associated with coronary heart disease, but not with metabolic diseases [18]. Like these authors we also noted no association between metabolic diseases and symptomatic HOA.

These results also found a significant association between the sum of the KL score and symptomatic HOA and the number of affected joints and symptomatic HOA. A systematic review of 16 studies showed a positive association between radiographic HOA and hand pain [14]. Some of these studies showed that people with a higher radiographic hand OA score as well as an increased number of hand joints with radiographic OA were more likely to report hand pain [14]. Schaefer et al. obtained comparable findings, observing that for each incremental increase in the sum of the KL grade, individuals were 33–51% more likely to report pain, and a higher number of affected joints also correlated with increased pain reports [54]. These results highlight the importance of interpreting radiographs in the assessment of symptomatic hand OA. This polyarticular presentation in symptomatic HOA seems to suggest the presence of systemic pathophysiological mechanisms, similar to those of chronic inflammatory joint diseases.

Moreover, these findings highlight the potential relevance of weight loss for HOA management, in line with existing recommendations for hip and knee osteoarthritis [55, 56]. Dietary interventions, exercise programs, and their combination are among the most extensively studied weight management strategies for OA. Strong evidence supports that combined diet and exercise interventions are essential for effective weight control, pain reduction, and improvement in quality of life for individuals with OA and obesity [57–60]. Furthermore, adherence to a Mediterranean diet and Prudent diet has been associated with better OA-related outcomes compared to a Western dietary pattern or a low-fat diet [61–63]. Our study reinforces, as reported by Mathieu et al*.* [53], the increase in cardiovascular risk factors in HOA patients, and further highlights that, among these factors, central obesity appears to be the key factor associated with symptomatic disease. Moreover, these results suggest that weight management, through exercise and weight loss, may represent an important therapeutic strategy for managing pain in HOA. This approach could potentially be integrated into future management recommendations for hand osteoarthritis. Given the increasing prevalence of obesity, even among younger populations, early screening and targeted prevention strategies are essential to delaying the progression of symptomatic HOA.

This study has some limitations due to the lack of data. Specifically, we did not have radiographs of all participants' hands, which would have provided another comparator group, namely individuals without hand OA. Another limitation is the self-reporting of heart diseases, although the questions regarding heart diseases inquired about a doctor's diagnosis. Additionally, it was not possible to assess functional hand disability due to a lack of data on functional scales for several participants. The strengths of this study include the large sample size, the population-based sample and the clinical examination conducted on all participants with standardized anthropometric measurements.

In conclusion, the authors identified an association between central adiposity and symptomatic HOA, as well as between radiographic severity and symptomatic HOA. These findings suggest that adiposity plays a significant role in HOA pain, and that improving metabolic control could enhance HOA outcomes. Additionally, the assessment of radiographic changes may aid in enhancing the monitoring of disease symptoms. Future research should aim to elucidate the mechanisms underlying these associations and to better characterise obesity subtypes in HOA, thereby enhancing our understanding and management of the disease.

## Data Availability

Data are available from the corresponding author upon reasonable request. Proposals must receive approval from the EPIPorto Data Access and Publications Committee. Scientific Coordinator: hbarros@med.up.pt.
